# Circ_0000888 regulates osteogenic differentiation of periosteal mesenchymal stem cells in congenital pseudarthrosis of the tibia

**DOI:** 10.1016/j.isci.2023.107923

**Published:** 2023-09-14

**Authors:** Zhuoyang Li, Yaoxi Liu, Yiyong Huang, Qian Tan, Haibo Mei, Guanghui Zhu, Kun Liu, Ge Yang

**Affiliations:** 1 Department of Orthopedics, Hunan Provincial Key Laboratory of Pediatric Orthopedics, Hunan Children’s Hospital, Changsha, Hunan, China; 2 Department of Orthopedics, the First Affiliated Hospital, College of Medicine, Zhejiang University, Hangzhou, Zhejiang, China

**Keywords:** Pathophysiology, Molecular biology, Cell biology, Stem cells research

## Abstract

Congenital pseudarthrosis of the tibia (CPT) is a refractory condition characterized by the decreased osteogenic ability in tibial pseudarthrosis repair. Periosteal mesenchymal stem cells (PMSCs) are multipotent cells involved in bone formation regulation. However, the mechanisms underlying its role in CPT remain unclear. In this study, we observed downregulation of circ_0000888 and pleiotrophin (PTN), as well as upregulation of miR-338-3p in CPT derived PMSCs (CPT-dPMSCs). Our results demonstrated that circ_0000888 and PTN likely enhanced the viability, proliferation, and osteogenic ability of PMSCs, while miR-338-3p had the opposite effect. Further analysis confirmed the regulatory relationship circ_0000888 suppressed the activity of miR-338-3p and upregulated the expression of its downstream target PTN by binding to miR-338-3p, consequently promoting the viability and osteogenic differentiation ability of CPT-dPMSCs. Our findings unveil an unexpected link between circ_0000888/miR-338-3p/PTN in promoting osteogenic ability and indicate the potential pathogenic mechanisms of CPT.

## Highlights

• CPT-dPMSCs present a significantly weakened osteogenic differentiation ability
• The expression of circ_0000888 decreased in CPT-dPMSCs
• Circ_0000888 exerts its effects by regulating targets miR-338-3p and PTN

## Graphical abstract

**Figure:**
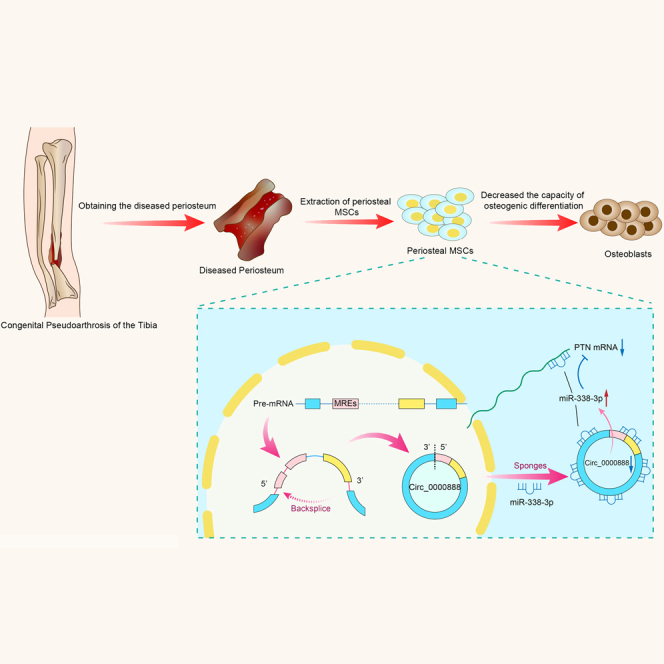

## Introduction

Congenital pseudarthrosis of the tibia (CPT) is a rare congenital disease primarily affecting children.^1^ It is characterized by developmental malformation of the tibia, narrowing of the medullary cavity, or fractures of the tibia, which ultimately result in the formation of a persistent and long-lasting pseudarthrosis.^2^ Despite being initially reported by Paget in 1891, the underlying pathogenesis of this disease remains unclear.^3^^,^^4^ Currently, surgical intervention is the mainstay treatment for CPT patients, however, the surgical procedure lack standardization.^5^ In our previous study, we identified a certain risk of complications associated with CPT, such as poor pseudarthrosis healing, recurrent fractures, and ankle valgus; consequently, patients encounter amplified treatment expenses and an augmented disease burden.^6^^,^^7^ Therefore, it is crucial to actively pursue research on the pathogenesis of CPT to explore more effective treatment options.

The periosteum, a connective tissue membrane that covers the surface of bone, plays a crucial role in bone growth, development, and healing of defects. It comprises two distinct layers, with the germinal layer harboring a profusion of mesenchymal stem cells (MSCs) exhibiting exceptional osteogenic differentiation capacity.^8^^,^^9^ Previous histological studies have indicated abnormal thickening of the periosteum at the lesion site of CPT,^10^ accompanied by the proliferation of fibrous tissue with hamartoma characteristics.^11^ Additionally, immunohistochemistry studies have shown that the pathological changes associated with CPT are primarily concentrated in the periosteum. Recent research has demonstrated that MSCs from the diseased periosteum in CPT patients, referred to as pathological MSCs, exhibit reduced osteogenic differentiation ability.^12^ Consequently, many scholars consider the diminished osteogenic differentiation ability of periosteal MSCs (PMSCs) to be a significant contributing factor in the development of CPT, although the precise molecular mechanism is still unclear.

MiRNAs, a class of non-coding RNAs, are ubiquitously present *in vivo*, typically ranging in length from 18 to 25 nucleotides. They predominantly bind to the 3′ untranslated region of target genes, thereby impeding translation or hastening degradation, thus exerting negative regulation on target gene expression in diverse biological processes.^13^ Extensive evidence highlights the crucial regulatory roles played by miRNAs in various physiological and pathological activities within the human body, encompassing cell differentiation, proliferation, apoptosis, cancer, and metabolic diseases.^14^^,^^15^ Additionally, an increasing body of research indicates that miRNAs play an equally critical role in modulating bone metabolism.^16^^,^^17^^,^^18^ Notably, elevated levels of miR-338-3p have been observed in osteoclast differentiation^19^ and its overexpression has been shown to promote osteoclast formation.^20^ Moreover, the overexpression of miR-338-3p in bone marrow MSCs significantly inhibits their osteogenic differentiation.^21^ However, limited studies have investigated the role of miR-338-3p in CPT.

CircRNA, a class of non-coding RNA, is widespread in eukaryotic cells.^22^ Unlike linear RNA, the head and tail of circRNA combine to form a covalently closed loop structure, which can effectively resist degradation by exonucleases with good stability. Recently, numerous studies have underscored one of the most significant functions of circRNA, acting as molecular sponges to inhibit the activity of miRNAs.^23^^,^^24^ MiR-338-3p has been reported to bind to a variety of circRNAs.^25^^,^^26^ Based on this knowledge, we are eager to explore the upstream circRNAs of miR-338-3p in CPT derived PMSCs (CPT-dPMSCs) and elucidate the mechanisms underlying their interactions.

In our study, we conducted a comprehensive investigation into the regulatory role of circZNF559 (also referred to as circ_0000888 in the CircInteractome database), derived from the zinc finger protein gene (ZNF) in the osteogenic differentiation of CPT-dPMSCs.

## Results

### Upregulation of miR-338-3p in CPT-dPMSCs and its impact on cellular activity

**Figure 1:**
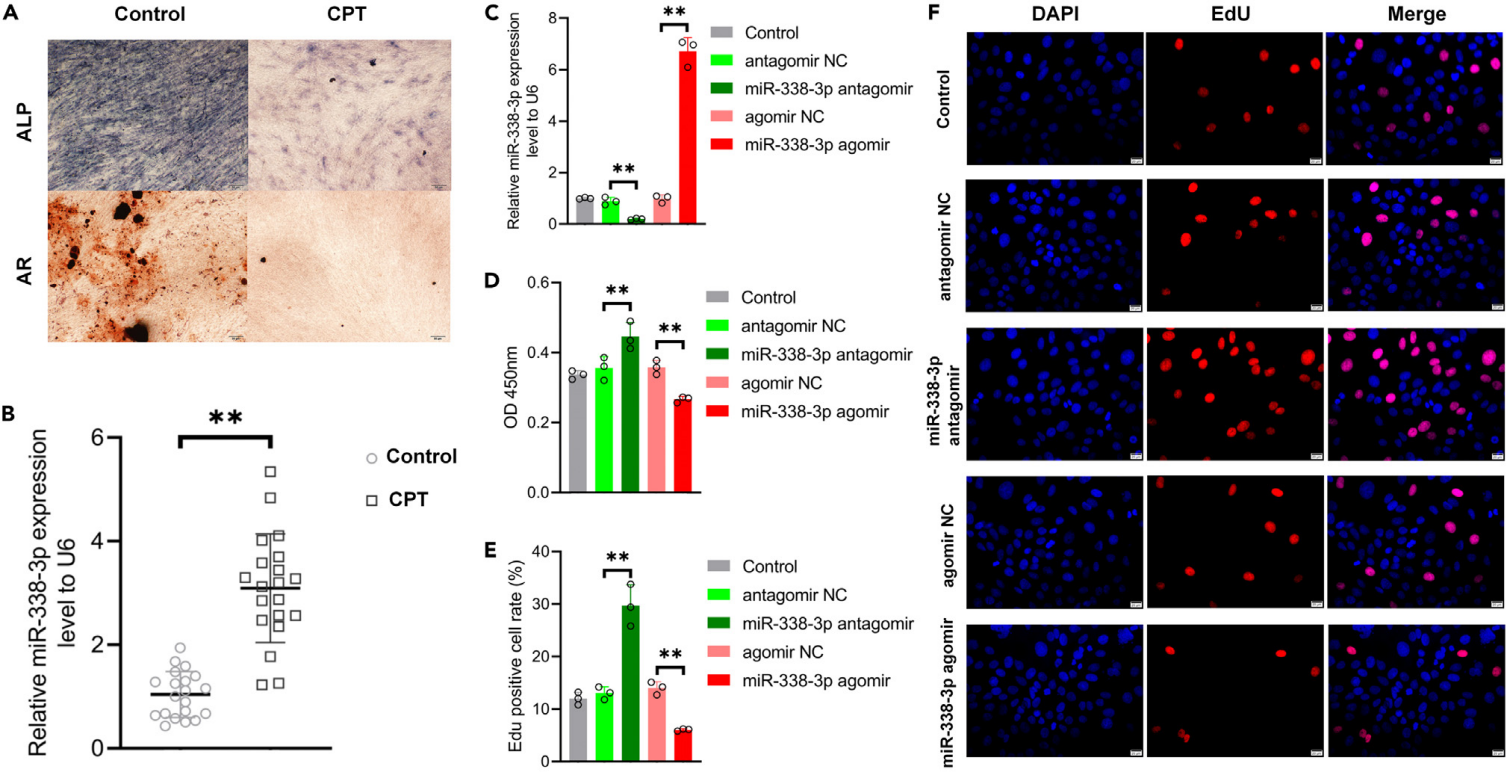
miR-338-3p was up-regulated in CPT-dPMSCs and involved in the regulation of the activity of PMSCs (A) ALP staining was used to detect the ALP level and AR staining was used to detect the calcium nodules deposition level in each group. Scale bar = 50 μm. (B) qRT-PCR analysis showed that the expression of miR-338-3p was significantly up-regulated in CPT-dPMSCs. ∗∗p *<* 0.01. (C) PMSCs were transfected with antagomir NC, miR-338-3p antagomir, agomir NC, and miR-338-3p agomir, and the expression level of miR-338-3p in PMSCs was verified by qRT-PCR. ∗∗p *<* 0.01. (D) CCK-8 assay was used to detect the cell viability of PMSCs in each group. ∗∗p *<* 0.01. (E) Edu assay was used to detect the proliferation of PMSCs in each group. ∗∗p *<* 0.01. (F) Fluorescence microscope photos of Edu staining. Blue fluorescence indicating total cells; red fluorescence indicating Edu positive cells. Scale bar = 20 μm. NC, negative control; OD, optical density.

To assess the effect of CPT on osteogenic differentiation, we performed osteogenic induction of differentiation in PMSCs from both the control group and CPT group. Our results confirmed a significant reduction in osteogenic differentiation ability in CPT-dPMSCs compared to the control group, as evidenced by decreased alkaline phosphatase (ALP) activity and decreased alizarin red (AR) staining (Figure 1A).

Furthermore, we examined the expression levels of miR-338-3p in both CPT-dPMSCs and control PMSCs using quantitative reverse transcription PCR (qRT-PCR). We observed a significant upregulation of miR-338-3p in CPT-dPMSCs compared to the control group (Figure 1B). To investigate the functional role of miR-338-3p in CPT-dPMSCs, we performed overexpression and repression experiments. The efficiency of miR-338-3p modulation was confirmed by qRT-PCR (Figure 1C).

Subsequently, we evaluated the impact of miR-338-3p on the viability and proliferation of CPT-dPMSCs using cell counting kit-8 (CCK-8) and 5-ethynyl-2′-deoxyuridine (EdU) assay, respectively. The results revealed that overexpression of miR-338-3p significantly suppressed cell viability and proliferation in CPT-dPMSCs. Conversely, inhibition of miR-338-3p expression reversed this effect (Figures 1D–1F). These findings indicate that miR-338-3p plays a critical role in inhibiting the activity of CPT-dPMSCs.

### Effect of miR-338-3p on osteogenic differentiation of CPT-dPMSCs

Further, we investigated the impact of miR-338-3p on the osteogenic differentiation ability of CPT-dPMSCs were detected by ALP staining, AR staining, and western blot analysis.

**Figure 2:**
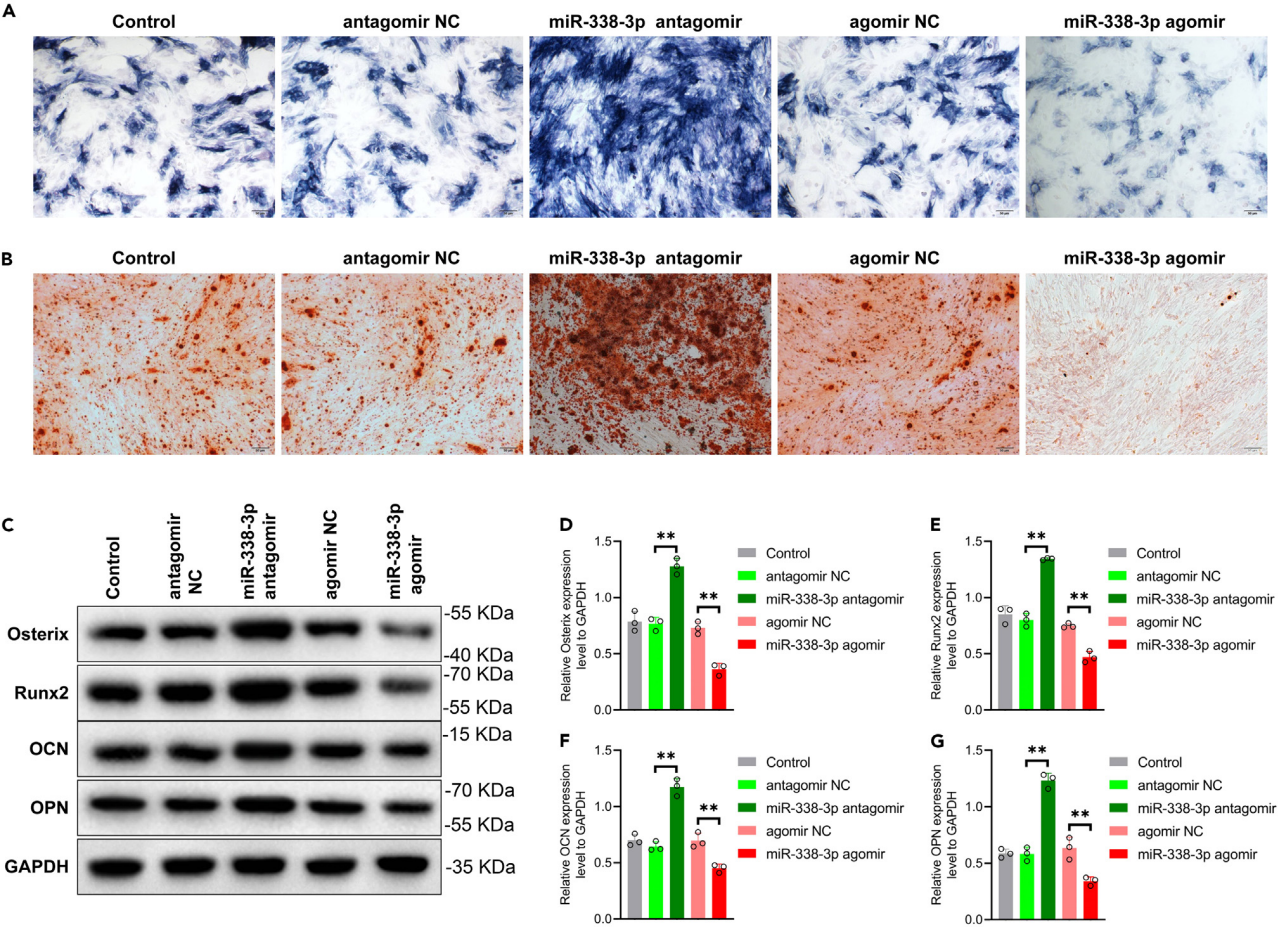
miR-338-3p significantly inhibited the osteogenic differentiation ability of PMSCs (A) ALP staining was used to detect ALP level of PMSCs transfected with antagomir NC, miR-338-3p antagomir, agomir NC and miR-338-3p agomir. Scale bar = 50 μm. (B) AR staining was used to detect the calcium nodule deposition level of PMSCs transfected with antagomir NC, miR-338-3p antagomir, agomir NC and miR-338-3p agomir. Scale bar = 50 μm. (C–G) Western blot analyzed the expression level of Osterix, Runx2, OCN and OPN in PMSCs transfected with antagomir NC, miR-338-3p antagomir, agomir NC and miR-338-3p agomir. ∗∗p *<* 0.01.

To evaluate the osteogenic potential, we examined the levels of ALP activity (Figure 2A) and calcium nodule formation (Figure 2B) during osteogenesis induction in CPT-dPMSCs. Our results demonstrated that increased expression of miR-338-3p significantly inhibited ALP levels and impaired calcium nodule formation, indicating suppressed osteogenic differentiation. Additionally, we analyzed the expression levels of osteogenesis-related proteins, including Osterix, runt-related transcription factor 2 (Runx2), osteocalcin (OCN), and osteopontin (OPN) through western blot analysis. Remarkably, we observed a substantial decrease in the expression of these proteins upon miR-338-3p overexpression (Figure 2C–2G). Conversely, inhibition of miR-338-3p expression yielded opposite results, promoting osteogenic differentiation in CPT-dPMSCs. These findings suggest that miR-338-3p negatively regulates the osteogenic differentiation of CPT-dPMSCs.

### miR-338-3p-mediated inhibition of pleiotrophin (PTN) expression reduces the activity and osteogenic differentiation of CPT-dPMSCs

**Figure 3:**
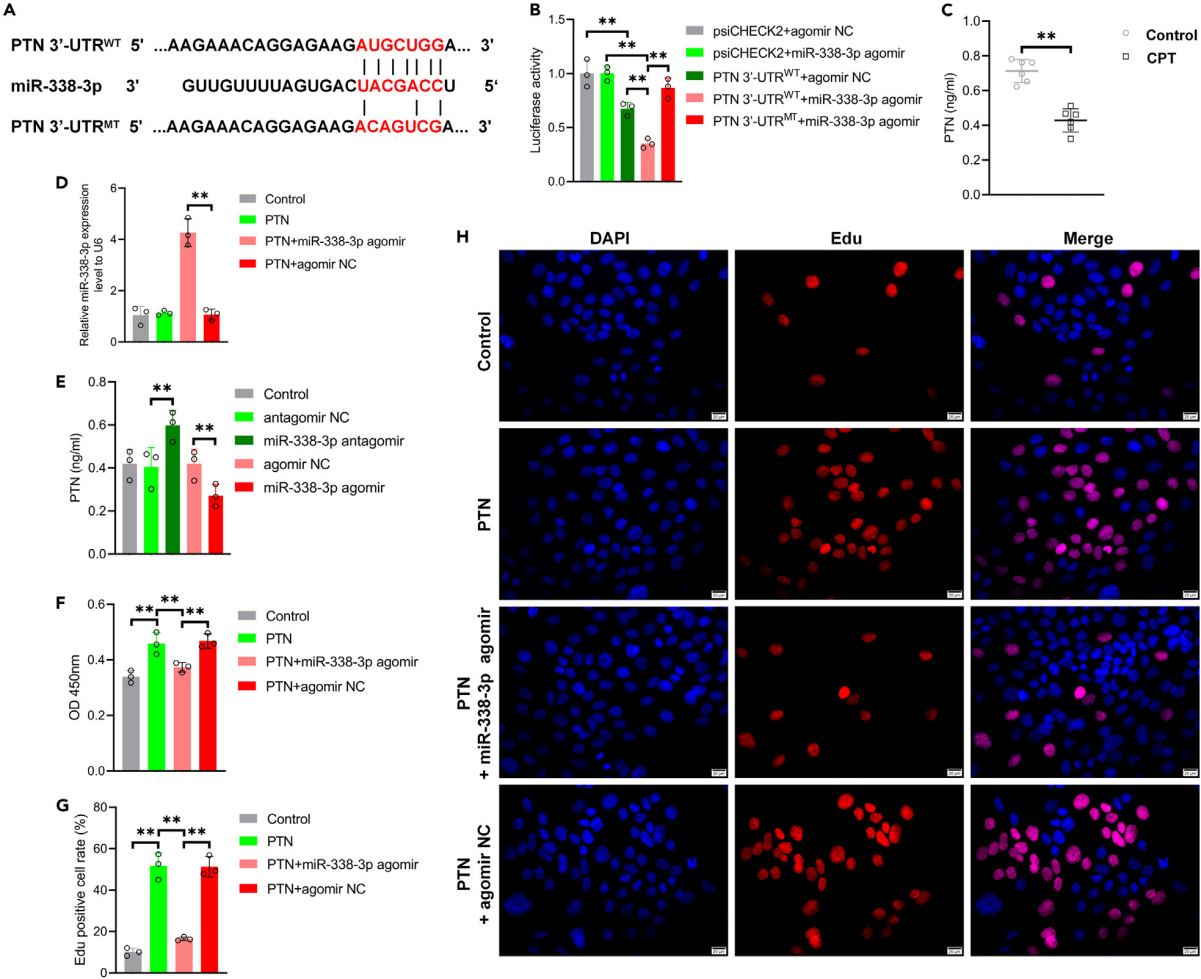
miR-338-3p affected the cell activity of PMSCs by targeting the downstream PTN (A) The 3 '-UTR region of PTN mRNA is the binding site of miR-338-3p. (B) agomir NC and miR-338-3p agomir were transfected into PMSCs, followed by transfection with luciferase constructs of wild-type PTN-3′UTR (3′UTR-WT) or mutant PTN-3′UTR (3′UTR-MT). Luciferase reporter gene assay showed that miR-338-3p could specifically reduce the luciferase activity of wild type reporter plasmid. ∗∗p *<* 0.01. (C) the expression of PTN was significantly down-regulated in CPT-dPMSCs. ∗∗p *<* 0.01. (D) PMSCs were transfected with agomir NC, and miR-338-3p agomir, and the expression level of miR-338-3p in PMSCs was verified by qRT-PCR. ∗∗p *<* 0.01. (E) qRT-PCR analyzed the expression level of PTN in PMSCs transfected with antagomir NC, miR-338-3p antagomir, agomir NC and miR-338-3p agomir. ∗∗p *<* 0.01. (F) CCK-8 assay was used to detect the cell viability of PMSCs treated with PTN and transfected with agomir NC and miR-338-3p agomir. ∗∗p *<* 0.01. (G) Edu assay was used to detect the proliferation of PMSCs treated with PTN and transfected with agomir NC and miR-338-3p agomir. ∗∗p *<* 0.01. (H) Fluorescence microscope photos of Edu staining. Blue fluorescence indicating total cells; red fluorescence indicating Edu positive cells. Scale bar = 20 μm.

Based on downstream target prediction analysis using miRBase, TargetScan, and other databases, PTN was identified as a potential downstream target of miR-338-3p (Table 1; Figure 3A). Luciferase reporter gene experiments confirmed that miR-338-3p inhibited the luciferase activity of the wild-type PTN gene, while the introduction of the 3′-UTR mutant PTN reversed this inhibitory effect (Figure 3B). Consequently, we examined the expression level of PTN in CPT-dPMSCs and observed a significant decrease compared to the control group, with an expression pattern opposite to that of miR-338-3p (Figure 3C). Moreover, we demonstrated that overexpression of miR-338-3p led to a significant downregulation of PTN expression, while inhibition of miR-338-3p increased PTN expression (Figures 3D and 3E). These findings collectively suggest that PTN is a downstream target of miR-338-3p, and miR-338-3p suppresses PTN expression by binding to its 3′-UTR and inhibiting its translation.

**Table 1:** The potential downstream target of miR-338-3p, related to Figure 3

miRNA Conserved sites	mRNA	Position in the UTR	Seed match	Context score	Context score percentile	Weighted context score	Conserved branch length
hsa-miR-338-3p	PTN	94–101	8mer	−0.51	99	−0.5	4.956

To assess the functional relevance of PTN and miR-338-3p in CPT-dPMSCs, we performed CCK-8 and Edu assays. The results demonstrated that increased PTN levels significantly enhanced the cell viability and proliferation ability of PMSCs. Conversely, overexpression of miR-338-3p attenuated the effects of PTN on CPT-dPMSCs (Figures 3F–3H). These findings indicate that miR-338-3p reduces the activity of CPT-dPMSCs by inhibiting PTN expression.

**Figure 4:**
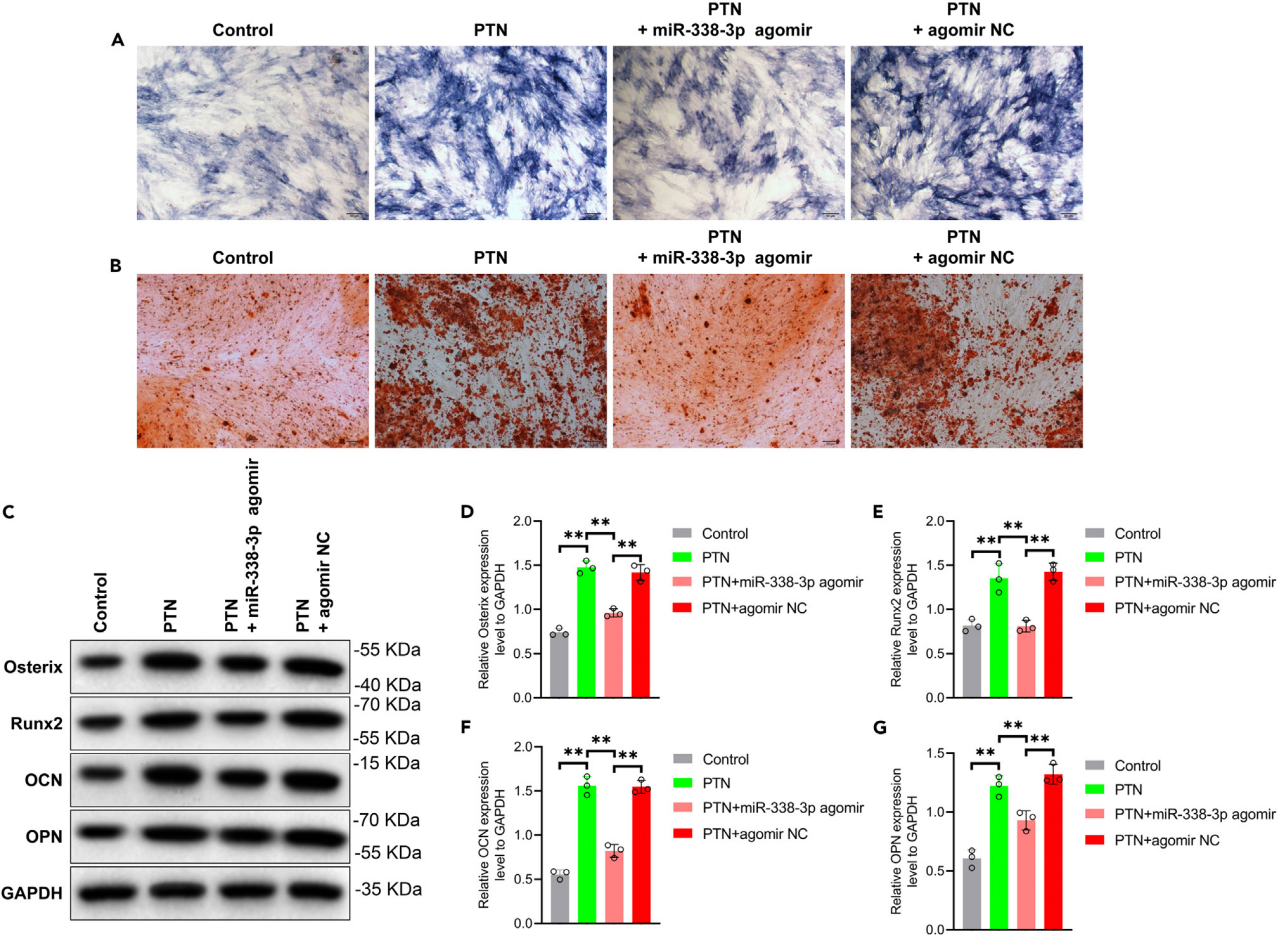
miR-338-3p affected the osteogenic differentiation ability of PMSCs by targeting PTN (A) ALP staining was used to detect ALP level of PMSCs treated with PTN and transfected with agomir NC and miR-338-3p agomir. Scale bar = 50 μm. (B) AR staining was used to detect the calcium nodule deposition level of PMSCs treated with PTN and transfected with agomir NC and miR-338-3p agomir. Scale bar = 50 μm. (C–G) Western blot analyzed the expression level of Osterix, Runx2, OCN and OPN in PMSCs treated with PTN and transfected with agomir NC and miR-338-3p agomir. ∗∗p *<* 0.01.

Furthermore, osteogenic differentiation was induced in different groups of PMSCs and evaluated through ALP staining, AR staining, and Western blot analysis. The results confirmed that PTN significantly increased ALP levels (Figure 4A) and calcium nodule formation (Figure 4B) during osteogenesis in CPT-dPMSCs. Additionally, the expression levels of osteogenesis-related proteins, including Osterix, Runx2, OCN and OPN, were significantly upregulated (Figures 4C–4G). In contrast, upregulation of miR-338-3p inhibited this bone-enhancing effect. These findings suggest that miR-338-3p inhibits the osteogenic differentiation of CPT-dPMSCs by suppressing PTN expression and interfering with the osteogenic differentiation process.

### Biological characteristics of Circ_0000888

**Figure 5:**
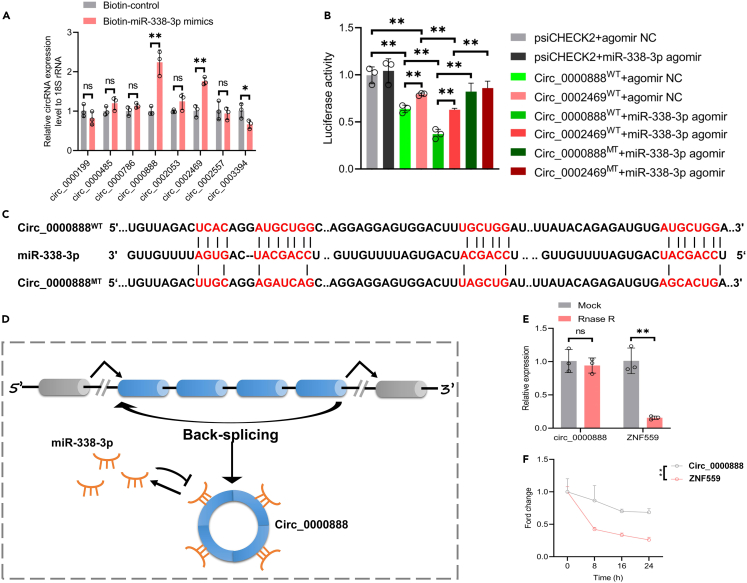
circ_0000888 acted as a molecular sponge of miR-338-3p (A) The interaction between the selected 8 circRNAs and miR-338-3p was detected by biotin-labeled miRNA pulldown assay. Among them, circ_0000888 and circ_0002469 had the stronger interaction. ∗∗p *<* 0.01. (B) Luciferase constructs of miR-338-3p agomir, circ_0000888 (WT/MT) and circ_0002469 (WT/MT) were transfected into PMSCs. Luciferase reporter gene experiment confirmed that the interaction between miR-338-3p and circ_0000888 was stronger. ∗∗p *<* 0.01. (C) circ_0000888 had four putative binding sites to miR-338-3p. (D) The diagram showed that circ_0000888 formed cyclic structure through back splicing and acted as a molecular sponge of miR-338-3p. (E) qRT-PCR was used to detect the expression levels of circ_0000888 and parental gene ZNF559 after RNase R digestion. ∗∗p *<* 0.01. (F) qRT-PCR was used to detect the expression levels of circ_0000888 and parental gene ZNF559 after 24 h of Actinomycin D treatment. ∗∗p *<* 0.01.

Based on miR-338-3p, we predicted and analyzed its possible interacting circRNAs by the CircInteractome database. The results clarified that miR-338-3p had binding sites to multiple circRNAs. We screened according to context score and context score percentile and removed circRNAs with sequences >3000nt, with ORFs, and without parental genes. We identified the 8 circRNAs most likely to interact as follows: circ_0000199, circ_0000485, circ_0000786, circ_0000888, circ_0002053, circ_0002469, circ_0002557 and circ_0003394 (Table 2). We then examined the expression levels of the above 8 circRNAs by biotin-labeled miRNA pulldown assay and selected 2 circRNAs that had the clear interaction relationship with miR-338-3p: circ_0000888 and circ_0002469 (Figure 5A). Further luciferase reporter gene experiments showed a stronger interaction between circ_0000888 and miR-338-3p (Figure 5B). We analyzed and compared the sequences of circ_0000888 and miR-338-3p by CircBase and other databases, and then noted that circ_0000888 had four potential binding sites to miR-338-3p (Figure 5C). These sites can act as molecular sponge sites for ceRNA (Figure 5D).

**Table 2:** the prediction of potential interacting circRNAs of miR-338-3p, related to Figure 5

miRNA mirbase ID	CircRNA mirbase ID	Site Type	Start	End	3′ pairing	Local AU	Position	TA	SPS	Context score	Context score percentile
hsa-miR-338-3p	hsa_circ_0000199	8mer - 1a	136	143	−0.039	−0.03	−0.08	0.019	−0.06	−0.437	99
hsa-miR-338-3p	hsa_circ_0000485	8mer - 1a	65	72	−0.008	−0.03	−0.098	0.019	−0.06	−0.424	99
hsa-miR-338-3p	hsa_circ_0000786	8mer - 1a	89	96	0.013	−0.005	−0.092	0.019	−0.06	−0.372	99
hsa-miR-338-3p	hsa_circ_0000888	8mer - 1a	405	412	0.003	0.018	−0.107	0.019	−0.06	−0.374	99
hsa-miR-338-3p	hsa_circ_0002053	8mer - 1a	55	62	0.034	−0.031	−0.1	0.019	−0.06	−0.385	99
hsa-miR-338-3p	hsa_circ_0002469	8mer - 1a	114	121	0.003	−0.006	−0.085	0.019	−0.06	−0.376	99
hsa-miR-338-3p	hsa_circ_0002557	8mer - 1a	159	166	−0.071	0.048	−0.107	0.019	−0.06	−0.418	99
hsa-miR-338-3p	hsa_circ_0003394	8mer - 1a	134	141	0.003	−0.029	−0.08	0.019	−0.06	−0.394	99

We further characterized the biological characteristics of circ_0000888: the UCSC Genome Browse database showed that human circ_0000888 is located in genome chr19: 9448471–9449995; the parental gene is ZNF559; it is relatively conserved among several species including rhesus monkey, dog, and elephant (Figure S1). Meanwhile, the expression levels of circ_0000888 and parental gene ZNF559 detected by qRT-PCR after RNase R digestion showed that circ_0000888 had good resistance to RNase R digestion (Figure 5E); actinomycin D assay showed that circ_0000888 had good stability (Figure 5F).

### Circ_0000888 achieves its biological function by targeting miR-338-3p and PTN

**Figure 6:**
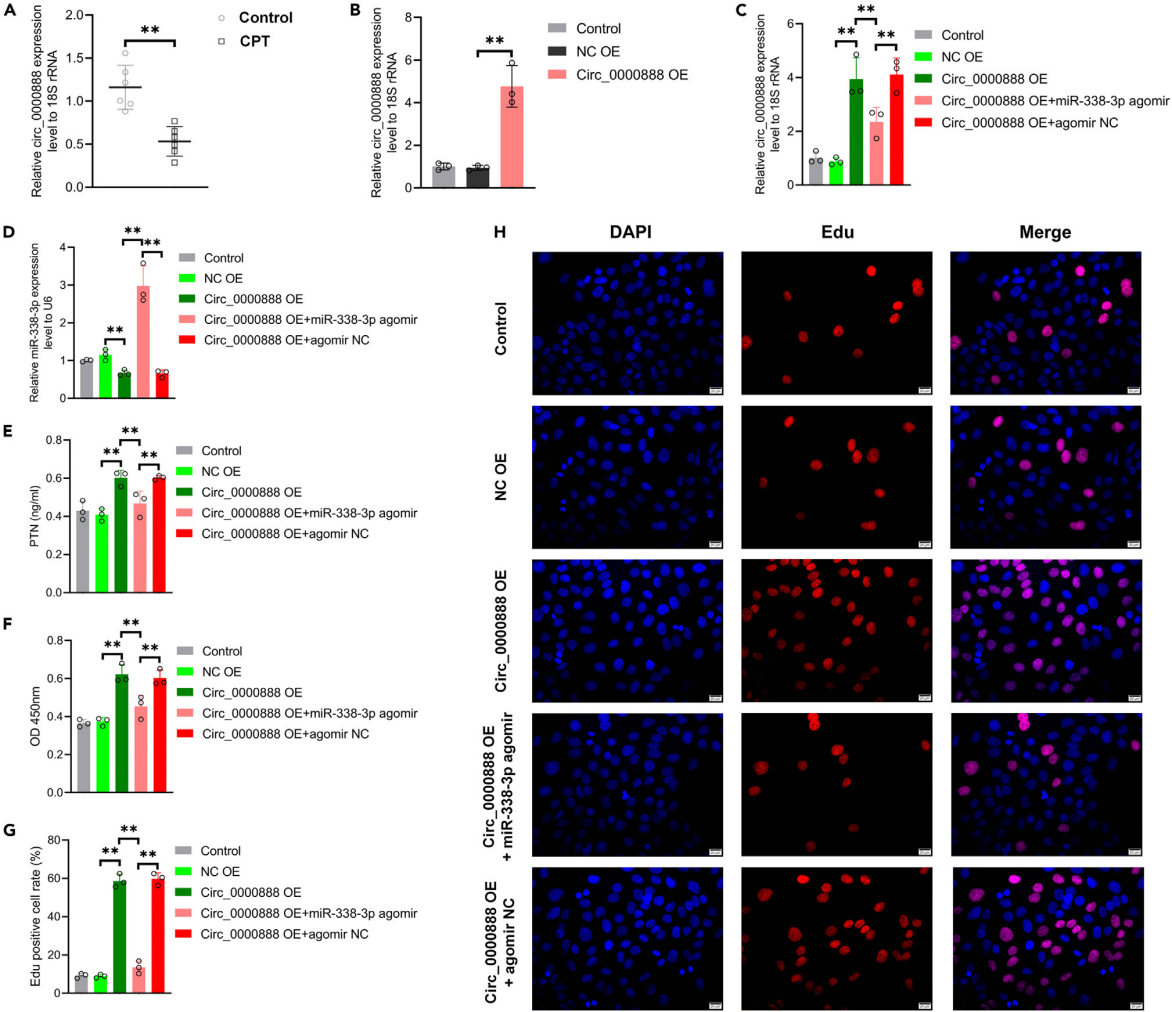
circ_0000888 affected the cell activity of PMSCs by targeting miR-338-3p and PTN (A) qRT-PCR analysis showed that the expression of circ_0000888 was significantly down-regulated in CPT-dPMSCs. ∗∗p *<* 0.01. (B) PMSCs were transfected with agomir NC and circ_0000888 agomir, and the expression level of circ_0000888 in PMSCs was verified by qRT-PCR. ∗∗p *<* 0.01. (C–E) agomir NC and circ_0000888 agomir were transfected into PMSCs, followed by transfection with miR-338-3p agomir. qRT-PCR was used to detect the expression levels of circ_0000888, miR-338-3p and PTN in each group. ∗∗p *<* 0.01. (F) CCK-8 assay was used to detect the cell viability of PMSCs transfected with agomir NC, miR-338-3p agomir and circ_0000888 agomir. ∗∗p *<* 0.01. (G) Edu assay was used to detect the proliferation of PMSCs transfected with agomir NC, miR-338-3p agomir and circ_0000888 agomir. ∗∗p *<* 0.01. (H) Fluorescence microscope photos of Edu staining. Blue fluorescence indicating total cells; red fluorescence indicating Edu positive cells. Scale bar = 20 μm.

The expression level of circ_0000888 in CPT-dPMSCs was detected by qRT-PCR and found to be significantly decreased with the opposite trend of miR-338-3p (Figure 6A). The circ_0000888 overexpression lentivirus was further constructed to infect PMSCs, and qRT-PCR verified the effect of circ_0000888 overexpression vector (Figure 6B), which proved that the overexpression lentivirus resulted in a significant increase in circ_0000888 levels in PMSCs, thereby inhibiting the expression of miR-338-3p and significantly increasing the expression of PTN; while on the basis of circ_0000888 overexpression, transfection of miR-338-3p overexpression lentivirus reversed the effect of circ_0000888 (Figures 6C–6E).

**Figure 7:**
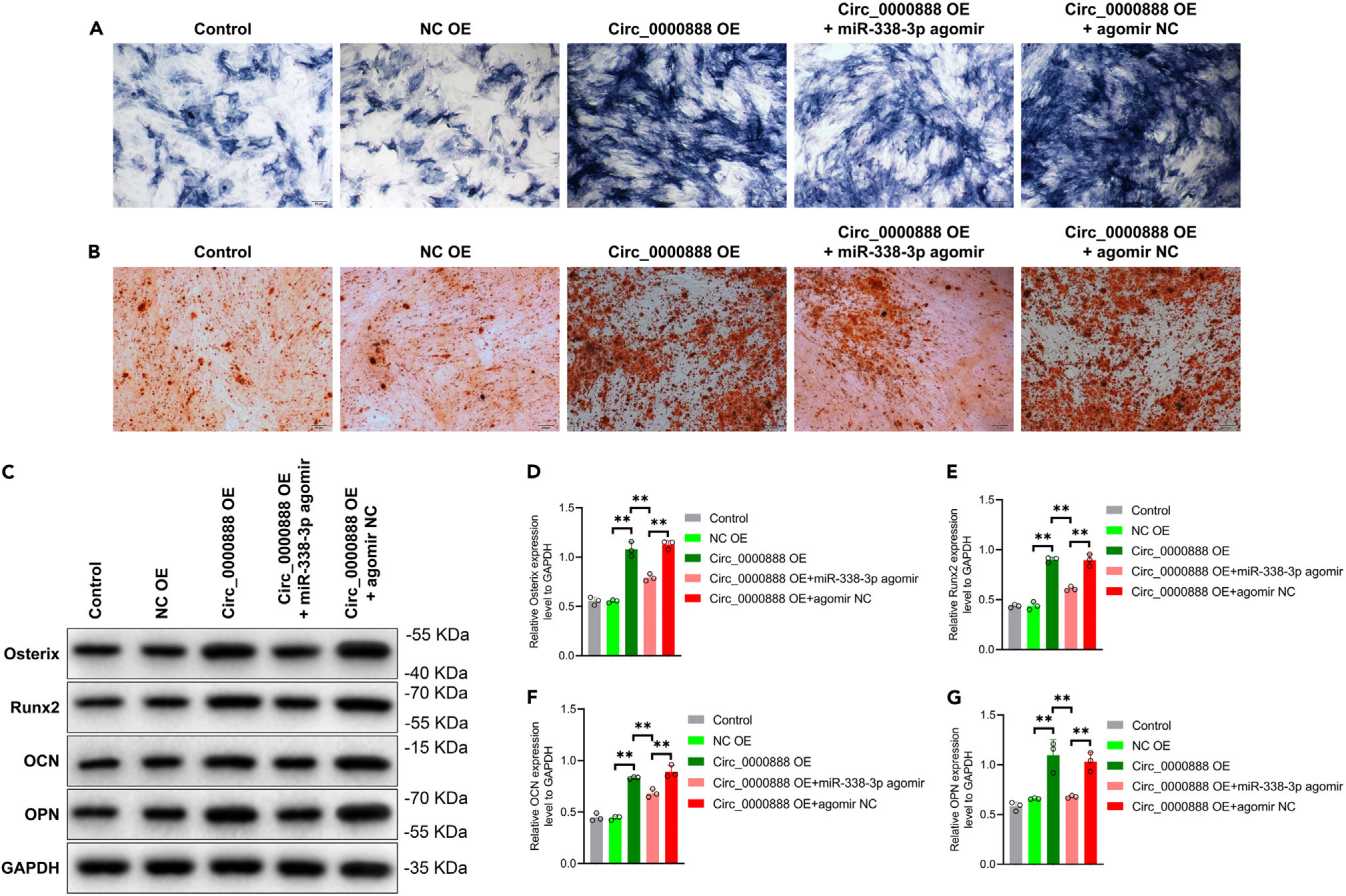
circ_0000888 affected the osteogenic differentiation ability of PMSCs by targeting miR-338-3p and PTN (A) ALP staining was used to detect ALP level of PMSCs transfected with agomir NC, miR-338-3p agomir and circ_0000888 agomir. Scale bar = 50 μm. (B) AR staining was used to detect the calcium nodule deposition level of PMSCs transfected with agomir NC, miR-338-3p agomir and circ_0000888 agomir. Scale bar = 50 μm. (C–G) Western blot analyzed the expression level of Osterix, Runx2, OCN and OPN in PMSCs transfected with agomir NC, miR-338-3p agomir and circ_0000888 agomir. ∗∗p *<* 0.01.

Meanwhile, the cell viability and proliferation ability of PMSCs were detected by CCK-8 and Edu assay, and the results showed that overexpression of circ_0000888 could significantly improve the cell viability and proliferation ability of PMSCs. Overexpression of miR-338-3p effectively reversed its function of enhancing cellular activity (Figures 6F–6H). Further, osteogenesis was induced in different groups of PMSCs and detected by ALP, AR staining, and western blot, which demonstrated that overexpression of circ_0000888 significantly increased the levels of ALP (Figure 7A) and calcium nodule formation (Figure 7B) and promoted the expression of osteogenic-related proteins during the osteogenic induction, while overexpression of miR-338-3p interrupted the osteogenic effect of circ_0000888 (Figures 7C–7G). The results of the present study suggest that circ_0000888 enhances cell activity and osteogenic differentiation ability in PMSCs through binding to inhibit miR-338-3p, where the decreased level of circ_0000888 is an important reason for the diminished cell activity and differentiation ability of CPT-PMSCs.

### Overexpression of Circ_0000888 can promote osteogenic activity *in* vivo

**Figure 8:**
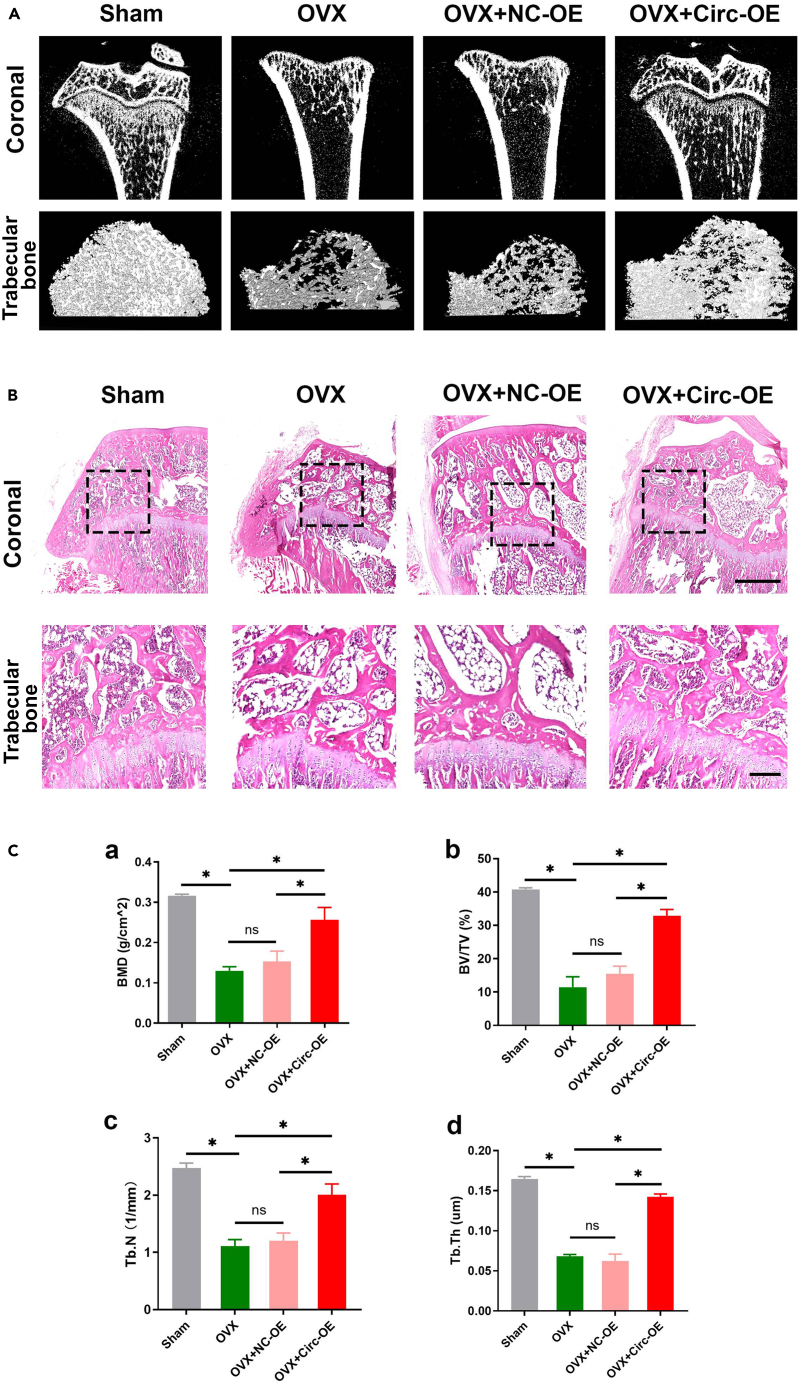
Overexpression of circ_0000888 promoted osteogenesis *in vivo* The rats were randomly divided into four groups: sham, OVX, OVX+NC-OE, OVX+Circ_0000888-OE groups. NC-OE and Circ_0000888-OE lentiviral vectors were injected into rats by caudal vein. (A) microCT images showed the bone structure of tibia. (B) HE staining slices showed bone trabecular tissue of tibia. (C) the parameters of tibial bone structure were as follows: a, BMD of the tibial was measured by dual-energy X-ray; b-d, area, number, and thickness of bone trabeculae were measured by microCT. ∗p *<* 0.05. OE, overexpression; BV/TV, bone volume fraction; Tb.N, trabecular number; Tb.Th, trabecular thickness.

To investigate whether circ_0000888 promotes osteogenic activity *in vivo*, we injected both overexpression and negative lentiviral vectors of circ_0000888 into rats. Our results demonstrated a significant decrease in bone mineral density (BMD), loss of bone mass, damaged bone tissue structure, and reduced quantity and quality of bone trabeculae in the ovariectomy (OVX) rats (p < 0.05), indicating the effectiveness of our model (Figure 8A).

After overexpressing circ_0000888, we conducted hematoxylin and eosin (HE) staining, which revealed an improvement in histological morphology: clearer bone trabeculae were observed, and their numbers had increased compared to the OVX group (Figure 8B). MicroCT analysis further confirmed that overexpression of circ_0000888 significantly enhanced the bone tissue structure of OVX rats by increasing the number, thickness, and area of bone trabeculae (p < 0.05) (Figure 8C). These findings suggested that overexpression of circ_0000888 promotes osteogenic activity *in vivo*.

## Discussion

In this study, we have identified circ_0000888 as an upstream target of miR-338-3p and characterized its downstream target gene PTN. Our investigation has shed light on the significant roles of circ_0000888, miR-338-3p, and PTN in the progression of CPT. Our findings demonstrate that circ_0000888 acts as a molecular sponge, sequestering miR-338-3p and attenuating its activity. Moreover, we propose that circ_0000888 exerts its functional effects by enhancing cell viability and promoting osteogenic differentiation in PMSCs through the inhibition of miR-338-3p and the upregulation of PTN expression. The decreased expression of circ_0000888 in CPT-dPMSCs appears to be an important contributing factor in the pathogenesis of the disease.

The balance of bone metabolism is primarily regulated by the interplay between osteoblasts and osteoclasts. Osteoblasts, which originate from MSCs, play a crucial role in bone formation and remodeling. In our study, we observed a significantly weakened osteogenic differentiation ability of CPT-dPMSCs compared to normal cells during osteogenesis induction. Furthermore, our previous investigations have revealed an imbalance in bone metabolism within the diseased periosteum of CPT patients, characterized by reduced osteogenesis and increased resorption in comparison to normal tissues.^27^ *In vitro* experiments utilizing serum exosomes from CPT patients have shown their inhibitory effect on osteogenic differentiation and promotion of osteoclast differentiation, aligning with previous findings.^28^ Additionally, pathological changes such as thickening of intra-periosteal small vessel walls with luminal narrowing and occlusion,^29^ as well as microenvironmental alterations, are observed at the lesion site.^30^ Collectively, these findings highlight the decreased osteogenic differentiation ability of CPT-dPMSCs as a significant contributing factor to the development of CPT. Moreover, it is plausible that inhibitory signals are involved in preventing MSCs from differentiating into osteoblasts.

MiR-338-3p has been recognized for its significant role in regulating various physiological and pathological processes in the human body. It has been reported to exert inhibitory effects on osteosarcoma growth by targeting MEF2C,^31^ as well as regulate the JAK1/STAT3 pathway to inhibit the progression of renal cell carcinoma.^32^ Furthermore, miR-338-3p has been shown to promote cartilage apoptosis and extracellular matrix degradation induced by IL-1β.^33^ Considering these findings, it is plausible that miR-338-3p acts as a suppressor of bone metabolism.

In our study, we investigated the expression level of miR-338-3p in CPT-dPMSCs and found a significant increase in its expression compared to normal cells, which sparked our interest. Through the regulation of miR-338-3p expression, we observed that its overexpression markedly inhibited cell viability and osteogenic differentiation ability in PMSCs. It is reasonable to infer that the elevated expression of miR-338-3p is a crucial factor contributing to the reduced osteogenic potential of the periosteum in CPT lesions, thereby functioning as an inhibitor of osteogenic differentiation in PMSCs. This finding aligns with previous studies demonstrating that overexpression of miR-338-3p hinders osteoblast differentiation by targeting Runx2 and Fgfr2, thereby inhibiting the expression of the osteogenic marker Osterix.^19^^,^^21^

Extensive research has demonstrated the crucial involvement of circRNAs in bone metabolism-related diseases.^34^^,^^35^^,^^36^ For instance, circ_0005752 has been shown to promote the osteogenic differentiation of adipose-derived stem cells,^37^ while circRNA AFF4 activates the SMAD1/5 pathway to enhance the osteogenic differentiation of BMSCs.^38^ Notably, circRNAs possess the ability to act as "molecular sponges" by sequestering miRNAs and inhibiting their function.^15^^,^^16^ Given their unique splicing mechanism, circRNAs contain multiple miRNA response elements (MREs) that can bind to miRNAs and impede their activity, leading to the upregulation of target gene expression.^18^ In our study, we discovered circ_0000888 as an upstream target of miR-338-3p, which harbors multiple MRE sites that specifically bind to miR-338-3p, functioning as a molecular sponge to inhibit its activity. Notably, we observed a significant decrease in the expression level of circ_0000888 in CPT-dPMSCs compared to normal cells. This reduction weakens the inhibitory effect on miR-338-3p, which in turn may contribute to the decreased osteogenic differentiation ability of CPT-dPMSCs. Thus, the diminished expression of circ_0000888 emerges as a potential causal factor for the impaired osteogenic potential observed in CPT-dPMSCs.

In our study, we identified PTN as a downstream target of miR-338-3p in the context of CPT. PTN is a small cationic protein that has been extensively studied in various physiological processes such as tissue repair, tumors, neural regeneration, adipose differentiation, and Alzheimer’s disease.^39^^,^^40^^,^^41^ However, its role in CPT has not been previously investigated. Increasing evidence supports the significant involvement of PTN in skeletal biology, particularly in promoting osteoblast adhesion to the extracellular bone matrix through its carboxy-terminal structural domain.^42^ PTN has been found to be expressed in the cellular matrix substrate during bone formation, facilitating osteoblast recruitment and attachment and thereby promoting new bone formation.^43^^,^^44^ Despite these known functions of PTN in bone biology, its role in CPT remains unexplored until our study. Our findings demonstrated that PTN enhances the cell viability and osteogenic differentiation of PMSCs and is part of the ceRNA network involving circ_0000888/miR-338-3p. Specifically, miR-338-3p directly targets the 3′-UTR of PTN mRNA, leading to the inhibition of PTN translation and function. On the other hand, circ_0000888 acts as a sponge for miR-338-3p, thereby increasing the expression of PTN.

In summary, our study not only shed light on the specific pathogenesis of CPT but also elucidated a ceRNA network involving circ_0000888/miR-338-3p/PTN. This discovery expands our understanding of the molecular mechanisms underlying CPT and provides valuable insights for further research and potential therapeutic interventions.

### Limitations of the study

Indeed, there are certain limitations that should be acknowledged in this study. The first is the absence of a recognized animal model for CPT, which prevented the validation of the findings *in vivo*. Second, besides molecular sponge, there may be other mechanisms for circ_0000888 to regulate the osteogenic differentiation process of CPT-dPMSCs, such as direct binding to mRNA to affect protein expression, etc., which calls for further studies to fully comprehend the specific molecular mechanisms of circ_0000888. By addressing these limitations and conducting further studies, including *in vivo* experiments and exploring alternative mechanisms of action, the understanding of the role of circ_0000888, miR-338-3p, and PTN in CPT can be expanded, providing more comprehensive insights into the pathogenesis and potential therapeutic targets for CPT.

### Conclusion

Circ_0000888 can inhibit the activity of miR-338-3p and increase the expression of the downstream target PTN, so as to promote the cell viability and osteogenic differentiation ability of PMSCs. The significant downregulation of circ_0000888 in CPT-dPMSCs may be one of the factors in CPT pathogenesis, which offers a potential strategy for CPT treatment.

## STAR★Methods

### Key resources table

**Table:** 

REAGENT or RESOURCE	SOURCE	IDENTIFIER
**Antibodies**		

Runx2	Abcam	Cat#ab76956; RRID: AB_1565955
OCN	Abcam	Cat# ab133612; RRID: AB_2916173
OPN	Abcam	Cat# ab214050; RRID: AB_2894860
Osterix	Abcam	Cat# ab209484; RRID: AB_2892207
GAPDH	Abcam	Cat# ab8245; RRID: AB_2107448

**Chemicals, peptides, and recombinant proteins**		

Collagenase P	Roche	Cat#11213857001
Dispase II	Roche	Cat#04942078001
Bone induction medium	Wuxi PH	Cat#CTCC-Y001
AR dye solution	sigma	Cat#A5533
Trizol reagent	Invitrogen	Cat#15596018
DAPI solution	Beyotime	Cat#C1002
Lipofectamine 2000	Invitrogen	Cat#11668019
Hematoxylin	Sigma	Cat#H9627
Eosin	Sinopdrug	Cat#71014544

**Experimental models: Organisms/strains**		

Rats: ovariectomy model	Lianchuan Biological Co., LTD	N/A

**Other**		

Reagent ALP kit	Wuxi PH	Cat#CTCC-JD002
CCK-8 kit	Beyotime	Cat#C0037
EdU positive cell proportion analysis kit	Beyotime	Cat#C0075S
SYBR Green PCR Super Mix kit	VAZYME	Cat#Q111-02
EntiLink™ 1st Strand cDNA Synthesis Kit	ELK Biotechnology	Cat#EQ003
RNA extracted kit	Geosai Biologics	Cat#RNR07250

### Resource availability

#### Lead contact

Further information and requests for resources and reagents should be directed to and will be fulfilled by the lead contact Ge Yang (jiamen88@zju.edu.cn).

#### Materials availability

This study did not generate new unique reagents.

### Experimental model and study participant details

#### Animals

Twenty healthy 12-week-old rats obtained from Lianchuan Biological Co., LTD (Hangzhou, China). The rats were randomly divided into four groups: sham group, OVX group, OVX+NC-OE group, and OVX+Circ_0000888-OE group. Preconditioning was performed during the first week, followed by weekly injection of lentiviral vector via the tail vein for eight weeks starting on the second week. At nine weeks after the operation, the tibia was removed from the rats. Animals of both sexes were used for all studies. All animal protocols were approved by the Ethics Committee of Hunan Children's Hospital and the First Affiliated Hospital of Zhejiang University, School of Medicine.

### Method details

#### Clinical sample collection, PMSCs separation and identification

The clinical samples of this study were used for the experiment and obtained the consent of the Ethics Committee of Hunan Children's Hospital and the First Affiliated Hospital of Zhejiang University Medical College and the informed consent of the children's families. CPT group: 20 periosteal samples of tibial pseudarthrosis in CPT patients undergoing surgical treatment, and control group: 20 samples of normal children admitted for trauma, fracture and other reasons from January 2020 to September 2021, including 11 males and 9 females in CPT group, aged from 11 months to 3 years 2 months, with an average age of (25 ± 10.2) months; 12 males and 8 females in control group, aged from 1 year 4 months to 3 years, with an average age of (25.2 ± 9.4) months. There was no statistically difference in basic characteristic between the two groups.

After the periosteal tissue sample was cut to 1 mm^3^, it was digested at 37°C for 1h with Collagenase P (1 mg/mL, Roche, 11213857001) and Dispase II (2 mg/mL, Roche, 04942078001). After DMEM culture solution containing 2% FBS was added, the supernatant was removed by centrifugation, and DNase I was added for resuspension at 37°C for 5 min. DMEM culture solution containing 10% FBS was added, and the tissue was resuspended gently for 5-10 times. The suspension was filtered by a 70-μm cell sieve, and the filtrate was inoculated into a culture dish. When the cell confluence reached 90% - 100%, the cells were digested by trypsin for subculture, and 3-6 generations of cells were taken for experiment.

The PMSCs obtained were subjected to centrifugation at 1500 r/min for 10 min, followed by resuspension in PBS. Then, a 100 μL volume of cell suspension was extracted and incubated with 3 μL of distinct anti-human monoclonal fluorescence-labeled antibodies (CD31, CD34, CD44, CD90) in the dark at room temperature for 30 min. After washing thrice with PBS, the cells were mixed with 2 mL of PBS containing 1% NaN_3_, centrifuged, and the supernatant was discarded. The cells were then fixed by adding 200 μL of PBS containing 0.1% polyformaldehyde and mixing. Finally, the sample was analyzed using flow cytometry. (Figure S2).

#### Immunohistochemical staining

ALP staining: added 300 μL of 4% gelatin to each well, standed at room temperature for 1h, sucked out the gelatin, placed the orifice plate in the super clean table to dry with an air blower, pressed 1 × 10^4^ cells/well cell planking, incubated for static culture at 37°C, 5% CO_2_; On the second day, the original culture medium was removed and replaced with bone induction medium (Wuxi PH, CTCC-Y001). The fresh and preheated bone induction medium was replaced every 3 days. After induction, ALP was detected: 40 μL Reagent ALP kit A (Wuxi PH, CTCC-JD002) was added to 1 mL of reaction buffer solution. After mixing, added 40 μL reagent B to prepare reaction working solution; Removed the culture medium, added 2 mL PBS to wash it twice; Added 1 mL of fixative into each hole and fix it at 37°C for 30 min; Removed the fixed solution, and added the reaction solution prepared in the first step into each hole and dyed it at 37°C for 30 min; and then took photos under the microscope for observation.

AR staining: after cell osteogenesis induction, removed the culture medium in the plate, washed it with PBS for 1-2 times, and added 400 μL 70% ethanol per pore, fixed at room temperature for 1 h; Washed with deionized water 2-3 times; Added 300 μL 1% AR dye solution (sigma, A5533) per hole, incubated at 37°C for 30 min; Removed the reaction solution and added deionized water to terminate the reaction; Took photos under the microscope for observation.

#### CCK-8 and EdU assay

##### CCK-8 assay

CCK-8 kit (Beyotime, C0037) was used to detect the cell viability. First, cell expansion. When the cell grown to the logarithmic growth phase, adjusted the cell concentration to 1 × 10^5^ cells/mL, inoculated in the culture plate with 200 μL per well at 37°C and 5% CO_2_; Detected the absorbance value 72 h after transfection, and added 20 μL CCK-8 to each hole during detection, incubated at 37°C and 5% CO_2_ for 2 h in dark. The OD value was measured by the microplate reader at 450 nm wavelength.

##### EdU assay

EdU positive cell proportion analysis kit (Beyotime, C0075S) was used to detect the cell DNA replication. Appropriate number of cells were cultured in the plate; 20 μM EdU working solution preheated at 37°C was added to the plates with equal volume, and the final concentration of EdU was 10 μM;Continue to incubate for 2 h, removed the culture medium, added 1 mL of fixative, and fix at room temperature for 15 min; Removed the fixed solution, washed the cells with 1mL washing solution for 3 times per well, and then incubated them with 1 mL penetrating solution at room temperature for 10-15 min; Added 0.5 mL Click reaction solution and covered the sample evenly; Incubated in dark at room temperature for 30 min; and added 1mL 1 × 4,6-diamidino-2-phenylindole (DAPI) solution (Beyotime, C1002) for each hole, incubated in dark at room temperature for 10 min, and then took fluorescence photos for observation.

#### Western blot

Extracted protein from lysed cells, added SDS loading buffer, and then marker and 30 ug protein were separately injected into the electrophoresis device for electrophoresis and membrane transfer; 5% skimmed milk powder was used to seal the membrane at 37°C for 2 h; Diluted the antibody with blocking solution to a proper concentration, and incubated it at 4°C overnight; After the primary antibody (Runx2 antibody, Abcam, ab76956, 1:1000; OCN antibody, Abcam, ab133612, 1:1000; OPN antibody, Abcam, ab214050, 1:1000; GAPDH antibody, Abcam, ab8245, 1:1000; Osterix antibody, Abcam, ab209484, 1:1000) was incubated, placed the PVDF membrane into the second antibody solution (Goat Anti Rabbit IgG (H+L) HRP, affinity, S0001, 1:5000; Goat Anti Mouse IgG (H+L) HRP, affinity, S0002, 1:5000), slow shaking incubation for 2h at 37°C; Added an appropriate amount of ECL luminous solution to the membrane and used the integrated chemiluminescence instrument to take photos.

#### qRT-PCR

Cells were lysed for RNA extraction by Trizol reagent (Invitrogen, 15596018). The first strand of cDNA was synthesized by EntiLink™ 1st Strand cDNA Synthesis Kit (ELK Biotechnology, EQ003); qRT-PCR was performed on the ABI QuantStudio 6 Real-Time PCR instrument (Life technologies). Each sample was made into 3 double holes and SYBR Green PCR Super Mix kit (VAZYME, Q111-02) was used. The reaction conditions were 95°C (15 s), 60°C (60 s), 95°C (15 s), and 40 cycles. The quantitative method was 2^-ΔΔ^ CT method. The primer sequence is shown in the Table S1.

#### Cell transfection

Agomir NC, miR-338-3p Agomir, antagonir NC and miR-338-3p antagonir were purchased from Gemma gene and transferred into cells with Lipofectamine 2000 (Invitrogen, 11668019).

#### Luciferase reporter gene experiment

According to the binding site, vectors of circ_0000888, circ_0002469 and 3 '-UTR wild-type and mutant-type PTN were constructed and grouped; Mixed LipofectamineTM 2000 and plasmid mixture diluent and added the cell lysis buffer; Centrifuged the supernatant after full lysis for determination. Took appropriate amount of sea kidney luciferase detection substrate to prepare the working solution; Added firefly luciferase detection reagent, mixed well and measured relative light unit (RLU), with the report gene cell lysis buffer as the blank control; After completing the above determination of firefly luciferase, added the sea kidney luciferase detection working solution, mixed well and then measured RLU with firefly luciferase luc as internal reference; The RLU value obtained by the sea kidney luciferase was divided by the RLU value obtained by the firefly luciferase; Compared the activation degree of the target reporter gene among different samples according to the ratio obtained.

#### RNase R digestion experiment

Cells were lysed for total RNA extraction by Trizol reagent. The extracted total RNA was digested with reference to the kit (Geosai Biologics, RNR07250): 5 μg RNA and 3 μM RNase R were added to the 20 μL reaction system, reacted in the RNase R buffer at 37°C for 10 min, and then heated to inactivate the enzyme activity at 95°C. After RNA extraction, qRT-PCR was used for quantitative detection.

#### Actinomycin D digestion experiment

Cells in logarithmic growth phase were treated with 2 μg/mL Actinomycin D for 0, 8, 16 and 24 h. Cells were collected, and RNA was extracted by Trizol. Circ_0000888 and ZNF59 mRNA levels were detected by qRT-PCR.

#### Biotin-miR-338-3p dependent RNA pulldown experiment

Cells were collected. Added lysis buffer to lyse cells after PBS washing and freeze them at -80°C for subsequent experiments. Added RIP Washing buffer and synthetic biotin labeled probe and incubated them at room temperature for 1 h; Added magnetic bead suspension after mixing; The magnetic bead sealing liquid was washed off through the magnetic frame; Then added probe complex solution; Rotated and mixed at room temperature; Repeat washing through RIP Washing buffer for 6 times; Elution was carried out through 95% formamide and 10 mM EDTA solution; The aqueous phase was separated by centrifugation of phenol, chloroform and isoamyl alcohol; Added salt solution and glycogen sedimentation aid; Washed and precipitated by anhydrous ethanol; Added ultrapure water without RNase to dissolve RNA after the ethanol was completely volatilized, and the target gene level was detected by qRT-PCR.

#### OVX animal model

Twenty healthy 12-week-old rats obtained from Lianchuan Biological Co., LTD (Hangzhou, China). The rats were randomly divided into four groups: sham group, OVX group, OVX+NC-OE group, and OVX+Circ_0000888-OE group. Preconditioning was performed during the first week, followed by weekly injection of lentiviral vector via the tail vein for eight weeks starting on the second week. At nine weeks after the operation, the tibia was removed from the rats, microCT was used to measure the bone mineral density (BMD). trabecular thickness (Tb.Th), and trabecular number (Tb.N).

#### HE staining

The tibia was decalcified in a decalcification solution for 30 days, washed three times with PBS, dehydrated using a 75%-100% gradient alcohol, and treated with xylene for tissue transparency. The tissue was then embedded in paraffin at 60°C for one hour, repeated three times, and cut into slices with a thickness of 5 μm.

After washing the slices with distilled water, they were stained with hematoxylin (Sigma H9627) for 2 min and washed until the tissues turned blue-purple. Next, they were rinsed with distilled water for 15s, followed by staining with eosin (Sinopdrug 71014544) for 8s. The slices were then rinsed with distilled water, dehydrated with absolute ethanol, and sealed with neutral gum. Finally, the slices were observed under a microscope and photographed.

### Quantification and statistical analysis

#### Statistics

Each group of independent experiments was repeated at least three times, and the data was expressed as mean ± standard deviation. The differences between the two groups were compared by student’s *t* test and passed the post test. All statistics were calculated using SPSS 23.0. *P* < 0.05 was statistically significant.

## Data Availability

• All data reported in this paper will be shared by the lead contact upon request. • This paper does not report original code. • Any additional information required to reanalyze the data reported in this paper is available from the lead contact upon request. All authors have approved the experiments and all experiments conform to the relevant regulatory standards.

## References

[1] Hefti F., Bollini G., Dungl P., Fixsen J., Grill F., Ippolito E., Romanus B., Tudisco C., Wientroub S. (2000). Congenital pseudarthrosis of the tibia: history, etiology, classification, and epidemiologic data. J. Pediatr. Orthop. B.

[2] Guille J.T., Kumar S.J., Shah A. (1998). Spontaneous union of a congenital pseudarthrosis of the tibia after Syme amputation. Clin. Orthop. Relat. Res..

[3] Boyd H.B., Harold B. (1982). Pathology and natural history of congenital pseudarthrosis of the tibia. Clin. Orthop. Relat. Res..

[4] Pannier S. (2011). Congenital pseudarthrosis of the tibia. Orthop. Traumatol. Surg. Res..

[5] Paley D. (2019). Congenital pseudarthrosis of the tibia: biological and biomechanical considerations to achieve union and prevent refracture. J. Child. Orthop..

[6] Li Z., Yu H., Huang Y., Liu Y., Zhu G., Tan Q., Mei H., Yang G. (2022). Analysis of risk factors affecting union and refracture after combined surgery for congenital pseudarthrosis of the tibia: a retrospective study of 255 cases. Orphanet J. Rare Dis..

[7] Zhou Y., Tan Q., Liu K., Liu Y., Zhu G., Mei H., Yang G. (2022). Epidemiological and clinical characteristics of congenital pseudarthrosis of the tibia in China. Front. Pediatr..

[8] Lin Z., Fateh A., Salem D.M., Intini G. (2014). Periosteum: biology and applications in craniofacial bone regeneration. J. Dent. Res..

[9] Toscani D., Bolzoni M., Accardi F., Aversa F., Giuliani N. (2015). The osteoblastic niche in the context of multiple myeloma. Ann. N. Y. Acad. Sci..

[10] Ho C.I., Tae-Joon C., Ju M.H. (2011).

[11] Cho T.J., Seo J.B., Lee H.R., Yoo W.J., Chung C.Y., Choi I.H. (2008). Biologic Characteristics of Fibrous Hamartoma from Congenital Pseudarthrosis of the Tibia Associated with Neurofibromatosis Type 1. J. Bone Joint Surg. Am..

[12] Granchi D., Devescovi V., Baglio S.R., Magnani M., Donzelli O., Baldini N. (2012). A regenerative approach for bone repair in congenital pseudarthrosis of the tibia associated or not associated with type 1 neurofibromatosis: correlation between laboratory findings and clinical outcome. Cytotherapy.

[13] Zhang X., Liang H., Kourkoumelis N., Wu Z., Li G., Shang X. (2020). Comprehensive Analysis of lncRNA and miRNA Expression Profiles and ceRNA Network Construction in Osteoporosis. Calcif. Tissue Int..

[14] Meydan C., Shenhar-Tsarfaty S., Soreq H. (2016). MicroRNA Regulators of Anxiety and Metabolic Disorders. Trends Mol. Med..

[15] Tüfekci K.U., Oner M.G., Meuwissen R.L.J., Genç S. (2014). The role of microRNAs in human diseases. Methods Mol. Biol..

[16] Gao M., Zhang Z., Sun J., Li B., Li Y. (2022). The roles of circRNA-miRNA-mRNA networks in the development and treatment of osteoporosis. Front. Endocrinol..

[17] Gao Y., Patil S., Qian A. (2020). The Role of MicroRNAs in Bone Metabolism and Disease. Int. J. Mol. Sci..

[18] Taipaleenmäki H. (2018). Regulation of Bone Metabolism by microRNAs. Curr. Osteoporos. Rep..

[19] Sun Q., Zhang B., Zhu W., Wei W., Ma J., Tay F.R. (2019). A potential therapeutic target for regulating osteoporosis via suppression of osteoclast differentiation. J. Dent..

[20] Long T., Guo Z., Han L., Yuan X., Liu L., Jing W., Tian W., Zheng X.H., Tang W., Long J. (2018). Differential Expression Profiles of Circular RNAs During Osteogenic Differentiation of Mouse Adipose-Derived Stromal Cells. Calcif. Tissue Int..

[21] Liu H., Sun Q., Wan C., Li L., Zhang L., Chen Z. (2014). MicroRNA-338-3p regulates osteogenic differentiation of mouse bone marrow stromal stem cells by targeting Runx2 and Fgfr2. J. Cell. Physiol..

[22] Memczak S., Jens M., Elefsinioti A., Torti F., Krueger J., Rybak A., Maier L., Mackowiak S.D., Gregersen L.H., Munschauer M. (2013). Circular RNAs are a large class of animal RNAs with regulatory potency. Nature.

[23] Kulcheski F.R., Christoff A.P., Margis R. (2016). Circular RNAs are miRNA sponges and can be used as a new class of biomarker. J. Biotechnol..

[24] Rong Z., Xu J., Shi S., Tan Z., Meng Q., Hua J., Liu J., Zhang B., Wang W., Yu X., Liang C. (2021). Circular RNA in pancreatic cancer: a novel avenue for the roles of diagnosis and treatment. Theranostics.

[25] Zheng J., Lin Y., Tang F., Guo H., Yan L., Hu S., Wu H. (2021). Promotive Role of CircATRNL1 on Chondrogenic Differentiation of BMSCs Mediated by miR-338-3p. Arch. Med. Res..

[26] Luo Q., Guo F., Fu Q., Sui G. (2021). hsa_circ_0001018 promotes papillary thyroid cancer by facilitating cell survival, invasion, G/S cell cycle progression, and repressing cell apoptosis via crosstalk with miR-338-3p and SOX4. Molecular therapy. Nucleic Acids.

[27] Li Z., Mei H., Liu K., Yang G. (2023). Differential expression and effect analysis of lncRNA-mRNA in congenital pseudarthrosis of the tibia. Front. Genet..

[28] Yang G., Yu H., Liu Y., Ye W., Zhu G., Yan A., Tan Q., Mei H. (2021). Serum-derived exosomes from neurofibromatosis type 1 congenital tibial pseudarthrosis impaired bone by promoting osteoclastogenesis and inhibiting osteogenesis. Exp. Biol. Med..

[29] Carlier A., Brems H., Ashbourn J.M.A., Nica I., Legius E., Geris L. (2016). Capturing the wide variety of impaired fracture healing phenotypes in Neurofibromatosis Type 1 with eight key factors: A computational study. Sci. Rep..

[30] Granchi D., Devescovi V., Baglìo S.R., Leonardi E., Donzelli O., Magnani M., Stilli S., Giunti A., Baldini N. (2010). Biological basis for the use of autologous bone marrow stromal cells in the treatment of congenital pseudarthrosis of the tibia. Bone.

[31] Cheng S., Chen C., Wang L. (2022). Knockdown of circ_0026579 ameliorates lipopolysaccharide (bacterial origin)-induced inflammatory injury in bronchial epithelium cells by targeting miR-338-3p/TBL1XR1 axis. Transpl. Immunol..

[32] Qi Q., Sun Y., Yang Y., Liu Y. (2022). Circ_0000274 contributes to renal cell carcinoma progression by regulating miR-338-3p/NUCB2 axis and JAK1/STAT3 pathway. Transpl. Immunol..

[33] Shuai S., Cai Q., Ou Y. (2022). Circular RNA circ_0008365 regulates SOX9 by targeting miR-338-3p to inhibit IL-1β-induced chondrocyte apoptosis and extracellular matrix degradation. J. Orthop. Surg. Res..

[34] Yu L., Liu Y. (2019). circRNA_0016624 could sponge miR-98 to regulate BMP2 expression in postmenopausal osteoporosis. Biochem. Biophys. Res. Commun..

[35] Zhai N., Lu Y., Wang Y., Ren X., Han J. (2018). Circular RNAs and hereditary bone diseases. Intractable Rare Dis. Res..

[36] Wang J., Wang T., Zhang F., Zhang Y., Guo Y., Jiang X., Yang B. (2022). Roles of circular RNAs in osteogenic differentiation of bone marrow mesenchymal stem cells (Review). Mol. Med. Rep..

[37] Wang M., Huan Y., Li X., Li J., Lv G. (2021). RUNX3 derived hsa_circ_0005752 accelerates the osteogenic differentiation of adipose-derived stem cells via the miR-496/MDM2-p53 pathway. Regen. Ther..

[38] Liu C., Liu A.S., Zhong D., Wang C.G., Yu M., Zhang H.W., Xiao H., Liu J.H., Zhang J., Yin K. (2021). Circular RNA AFF4 modulates osteogenic differentiation in BM-MSCs by activating SMAD1/5 pathway through miR-135a-5p/FNDC5/Irisin axis. Cell Death Dis..

[39] Herradon G., Ramos-Alvarez M.P., Gramage E. (2019). Connecting Metainflammation and Neuroinflammation Through the PTN-MK-RPTPβ/ζ Axis: Relevance in Therapeutic Development. Front. Pharmacol..

[40] Sorrelle N., Dominguez A., Brekken R.A. (2017). From top to bottom: midkine and pleiotrophin as emerging players in immune regulation. J. Leukoc. Biol..

[41] Lamprou M., Kaspiris A., Panagiotopoulos E., Giannoudis P.V., Papadimitriou E. (2014). The role of pleiotrophin in bone repair. Injury.

[42] Gieffers C., Engelhardt W., Brenzel G., Matsuishi T., Frey J. (1993). Receptor binding of osteoblast-specific factor 1 (OSF-1/HB-GAM) to human osteosarcoma cells promotes cell attachment. Eur. J. Cell Biol..

[43] Imai S., Heino T.J., Hienola A., Kurata K., Büki K., Matsusue Y., Väänänen H.K., Rauvala H., Rauvala H. (2009). Osteocyte-derived HB-GAM (pleiotrophin) is associated with bone formation and mechanical loading. Bone.

[44] Fan J.B., Liu W., Yuan K., Zhu X.H., Xu D.W., Chen J.J., Cui Z.M. (2014). EGFR trans-activation mediates pleiotrophin-induced activation of Akt and Erk in cultured osteoblasts. Biochem. Biophys. Res. Commun..

