# Supplementary material for: Circ_0000888 regulates osteogenic differentiation of periosteal mesenchymal stem cells in congenital pseudarthrosis of the tibia
Source: iScience. 2023 Sep 14;26(10):107923. doi: 10.1016/j.isci.2023.107923 (PMC10551655; doi:10.1016/j.isci.2023.107923)
Supplement: Document S1. Figure S1 [file mmc1.pdf]

iScience, Volume *26*

Supplemental information

Circ_0000888 regulates osteogenic differentiation of periosteal mesenchymal stem cells in congenital pseudarthrosis of the tibia

Zhuoyang Li, Yaoxi Liu, Yiyong Huang, Qian Tan, Haibo Mei, Guanghui Zhu, Kun Liu, and Ge Yang

A
B
C
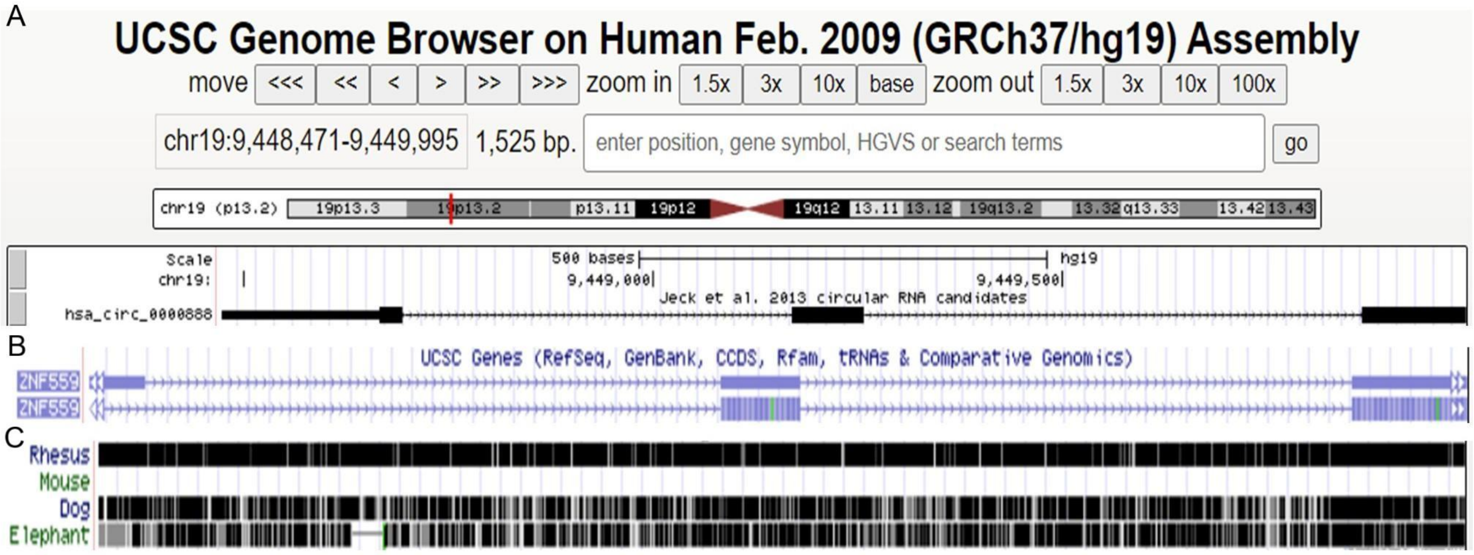

Supplementary Figure S1. Basic characteristics of Circ_0000888, related to Figure 5. A.) the genomic location of Circ_0000888. B.) ZNF559 as parent gene. C.) relatively conservative in more than one species.

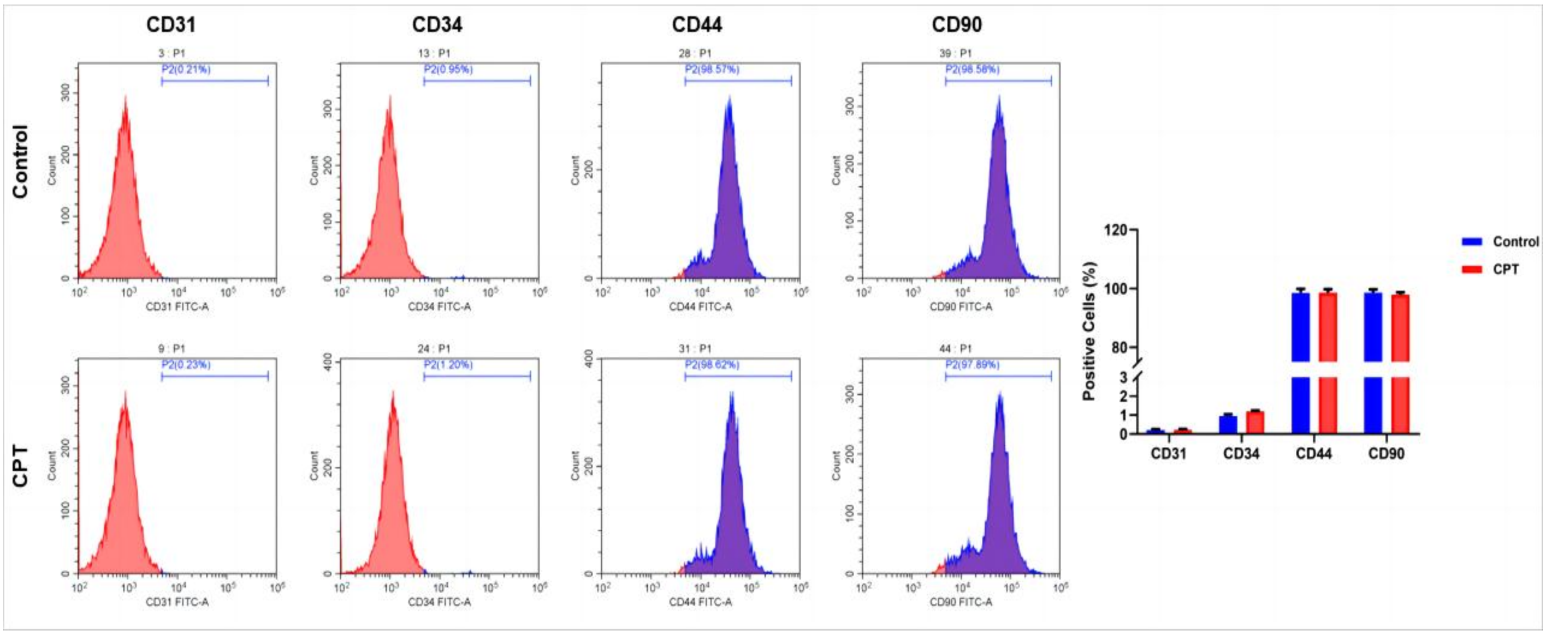

Supplementary Figure S2. Identification of PMSCs, related to STAR Methods. Flow cytometry was used to detect the positive ratio of molecular markers CD31, CD34, CD44 and CD90 in PMSCs from different sources. The results showed that the CD31 and CD34 cell positivity rates for identified cell lines were less than 0.5%, while the CD44 and CD90 cell positivity rates were higher than 98%.

Supplementary Table S1. The primer sequences, related to STAR Methods

Primer	Primer Sequence (5'-3')
miR-338-3p	GGTCCAGCATCAGTGA
	GAGCAGGCTGGAGAA
18s	AGGCGCGCAAATTACCCAATCC
	GCCCTCCAATTGTTCCTCGTTAAG
circ_0000199	CATTGCTTTCAGGGCTCTTGA
	CCGCTCTCTCGACAAATGGA
circ_0000485	AGCAGTGTGGTTCCTTTCCA
	CCAAGCAGCTTCTTTTTGTG
circ_0000786	TGGGGTACAGGTTATGCTGG
	GCTAGAAAACAGAGATGGTGCC
circ_0000888	CTTTGAGGATGTGGCTGTGG
	CTCATCTGTCATCAAGCGCC
circ_0002053	TGAGGAGGAGGAGATTGGGA
	CAGGTTAAGGCAGGACTGGA
circ_0002469	CCACGAACACAAATGCAGGA
	AGGTCAATCTGGGGTGGAAG
circ_0002557	CGACCCCAGGATCACGAC
	GGACACGAGTTTTCCTGAAGG
circ_0003394	ACCTCATGTTGCTCCTGTGA
	TGCAATGGAGCCAATAGAACG
U6	CGCTTCGGCAGCACATATAC
	AAATATGGAACGCTTCACGA
